# Reverse resistance to immune checkpoint inhibitor in a patient with recurrent cardia cancer by intratumoral injection of recombinant human adenovirus type 5: a case report and literature review

**DOI:** 10.3389/fonc.2024.1465664

**Published:** 2024-11-11

**Authors:** Qiu Zhao, Min Xiao, Jian Ma, Cong Fu, Qianqian Gao, Yanzhi Bi

**Affiliations:** ^1^ Department of Oncology, Changzhou Tumor Hospital Affiliated to Soochow University, Changzhou, China; ^2^ Department of Pathology, Changzhou Tumor Hospital Affiliated to Soochow University, Changzhou, China

**Keywords:** cardia cancer, immune checkpoint inhibitors, recombinant human adenovirus type 5, CD4+ T cell, CD8+ T cell, immune microenvironment

## Abstract

Advanced metastatic cardia cancer is an intractable malignance with poor prognosis. It is often accompanied by upper digestive tract obstruction, which seriously affects the quality of patients. Therefore, effective relief of eating obstruction is an important goal in the treatment of cardia cancer. Immune checkpoint inhibitors (ICIs) have shown significant efficacy in cardia cancer, but only a small percentage of patients will benefit from them due to immune resistance. Oncolytic viruses have been shown to enhance the efficacy of ICIs by altering the immune microenvironment. This indicates that oncolytic virus has the potential value of overcoming the immune resistance of cardia cancer. Here, we present a case with local recurrent and multiple metastatic cardia cancer accompanied by eating obstruction. After 4 cycles of chemotherapy plus ICI therapy, the patient´s metastases were significant shrink, but the recurrent carida lesion were almost unchanged. Then we implemented exploratory local injection of recombinant human adenovirus type 5(H101) into recurrent cardia lesion by painless gastroscopy. Surprisingly, the cardia lesion shrank significantly, and the eating obstruction was greatly relieved. We also observed a significant increase of infiltrated CD4+T cells in biopsy tissues after H101 treatment. Our study not only conformed the value of oncolytic viruses to reverse ICI resistance in patients with gastric cancer, but also revealed its underlying impact on immune microenvironment.

## Introduction

Advanced cardia cancer is a highly aggressive and heterogeneity malignance with a poor five-year survival rate (1). Patients are often suffer from eating obstruction, which can seriously affect the patient’s quality of life, which can seriously affect the patient’s quality of life, resulting in poor nutrition and even shorter survival. Therefore, it is a difficult and urgent problem to be solved in clinic practice. In recent years, immune checkpoint inhibitors (ICIs) have shown significant efficacy in cardia cancer, but only a small subgroup of patients will benefit from them due to immune resistance (2–5). Therefore, overcoming immune resistance is an imperative task.

Oncolytic viruses have been shown to enhance the efficacy of ICIs by directly lysing tumor cells and altering the immune microenvironment (6, 7). However, there are few reports about the effect of oncolytic viruses on reversing immune resistance of cardia cancer. We have a patient diagnosed with metastatic cardia cancer who underwent 4 cycles of immune checkpoint inhibitor combined with chemotherapy without no shrink in cardia lesions. What’s worse, the patient was suffered from the symptom of eating obstruction. However, the patient refused radiotherapy for fear of its toxic side effects. Considering the unique anti-tumor mechanism of oncolytic virus and its mild toxic side effects, we innovatively injected H101 into the cardia lesions by electronic gastroscope. Surprisingly, the cardia lesions were significantly reduced, and the parent symptom of eating obstruction were significantly relieved. The details are reported below.

## Case presentation

In April 2021, a 72-year-old man was diagnosed with cardia cancer and subsequently underwent radical gastrectomy. Pathological diagnosis reported a poorly differentiated ulcerative adenocarcinoma (stage ШB,T3N3M0). He received oral S-1 (40mg twice daily for 14 days, discontinued for 7 days, and repeated every 21 days) as adjuvant chemotherapy regimen for about 4 months, but discontinued due to significant gastrointestinal reaction.

**Figure 1:**
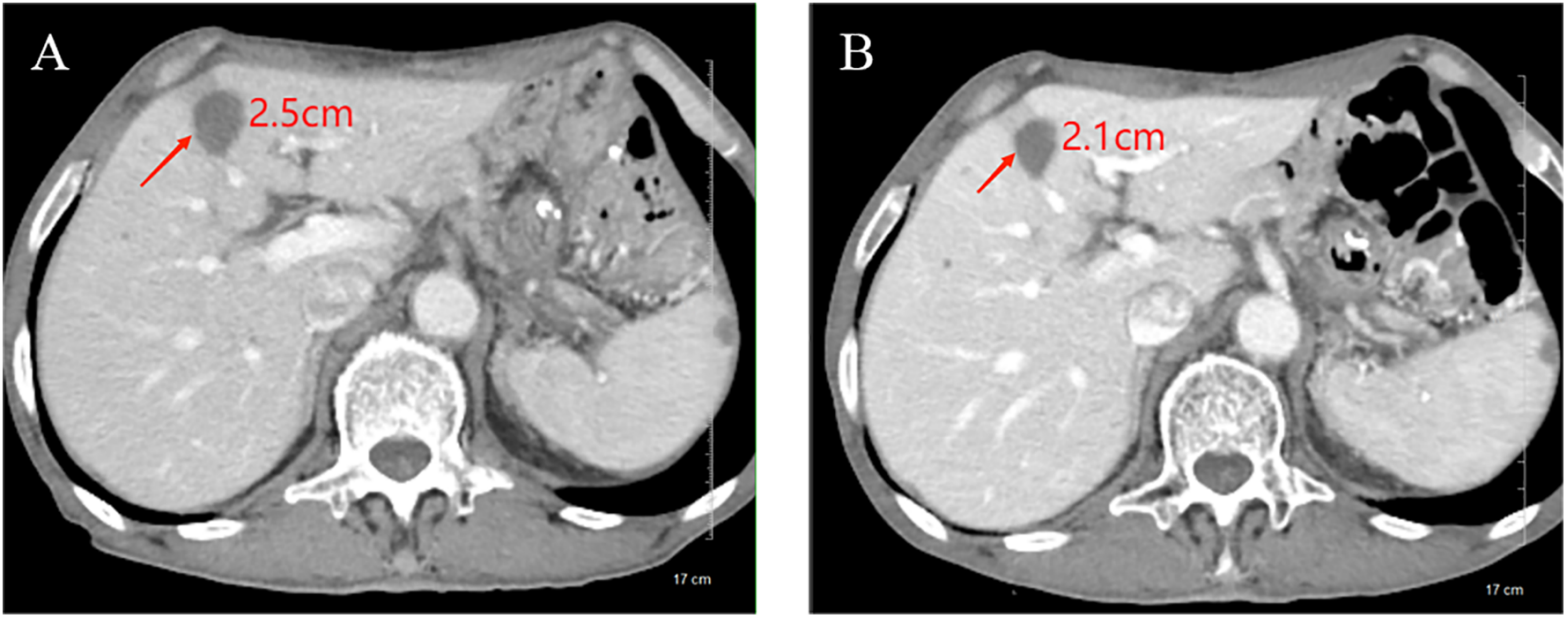
Comparison of longest diameter of liver metastases on CT images before and after chemotherapy plus ICI therapy. **(A)** Before treatment, the longest diameter of liver metastases was about 2.5cm; **(B)** After treatment, the longest diameter of liver metastases was reduced to 2.1cm.

**Figure 2:**
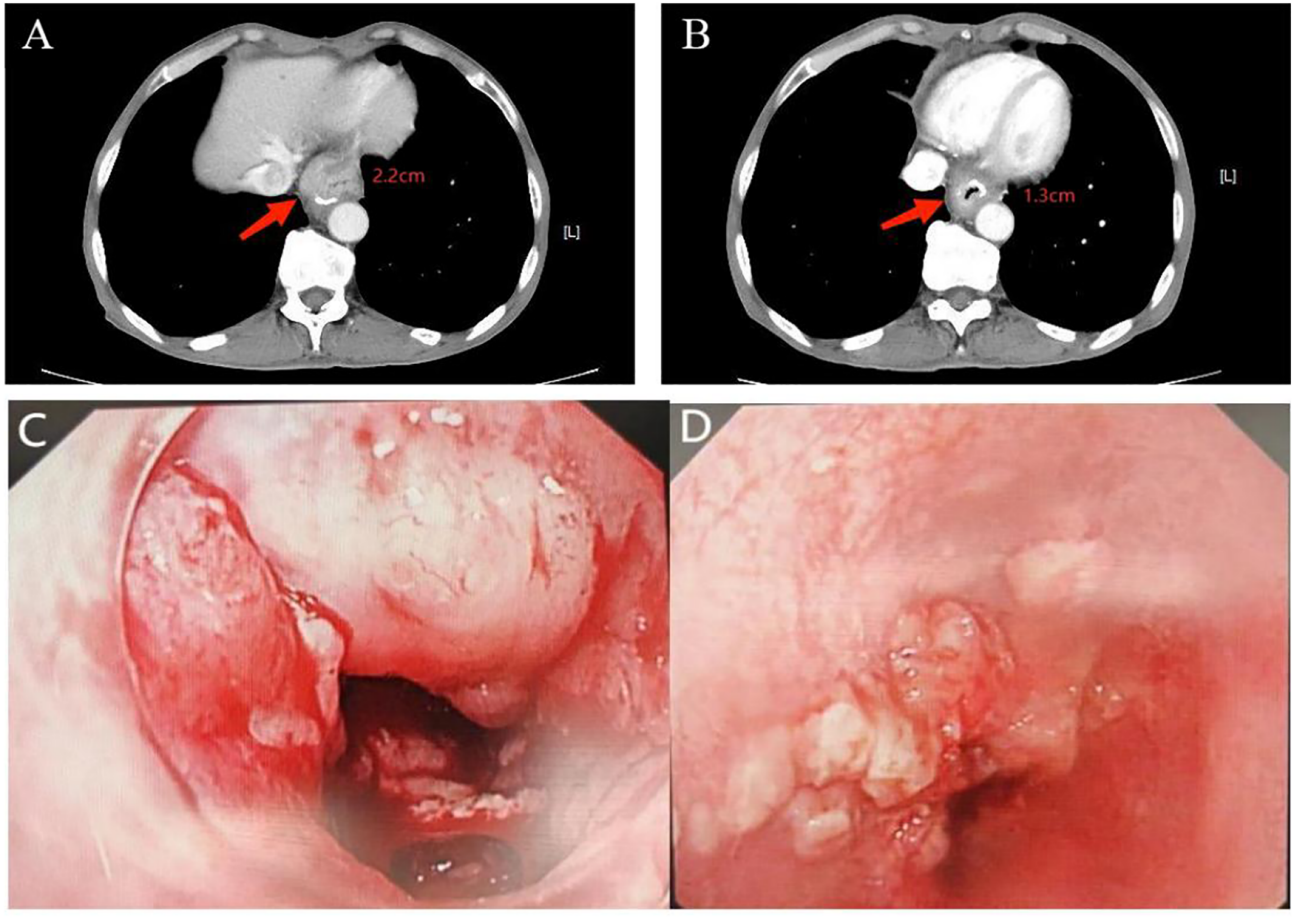
Comparison of cardia lesion thickness on CT and gastroscopy images before and after H101 treatment. **(A)** Before H101 treatment, the thickest part of the cardia lesion was about 2.2cm; **(B)** After H101 treatment, the thickest part was significantly reduced to 1.3cm; **(C)** Before H101 treatment, gastroscopy showed that the cardia lesions were large and protruding into the cavity; **(D)** After H101 treatment, gastroscopy showed that the cardia lesions were significantly reduced and the local stenosis was significantly improved.

**Figure 3:**
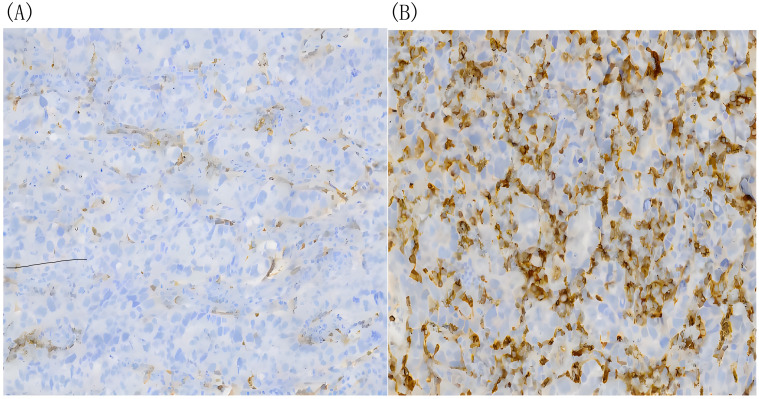
The density of infiltrating CD4+T cells tested by immunohistochemical DAB staining. **(A)** Before H101 treatment, the density of infiltrating CD4^+^ T cells in cardia lesion is low(-);**(B)** After H101 treatment, the density of infiltrating CD4^+^ T cells increased significantly(+++). Reagent information: Rabbit monoclonal to CD4, Product code: ab133616, Abcam (Shanghai) Trading Co. LTD. Quantitative method: The percentage of artificially counted positive cells in all cells of 10 times microscope field: Count the proportion of positive cells accurately located by IHC markers in the total cells and score (using nucleus as the cell localization standard). The standard is as follows: 25% of the total cell number is (-), 25%-50% is (+), 50-75% is (++), and more than 75% is (+++).

**Figure 4:**
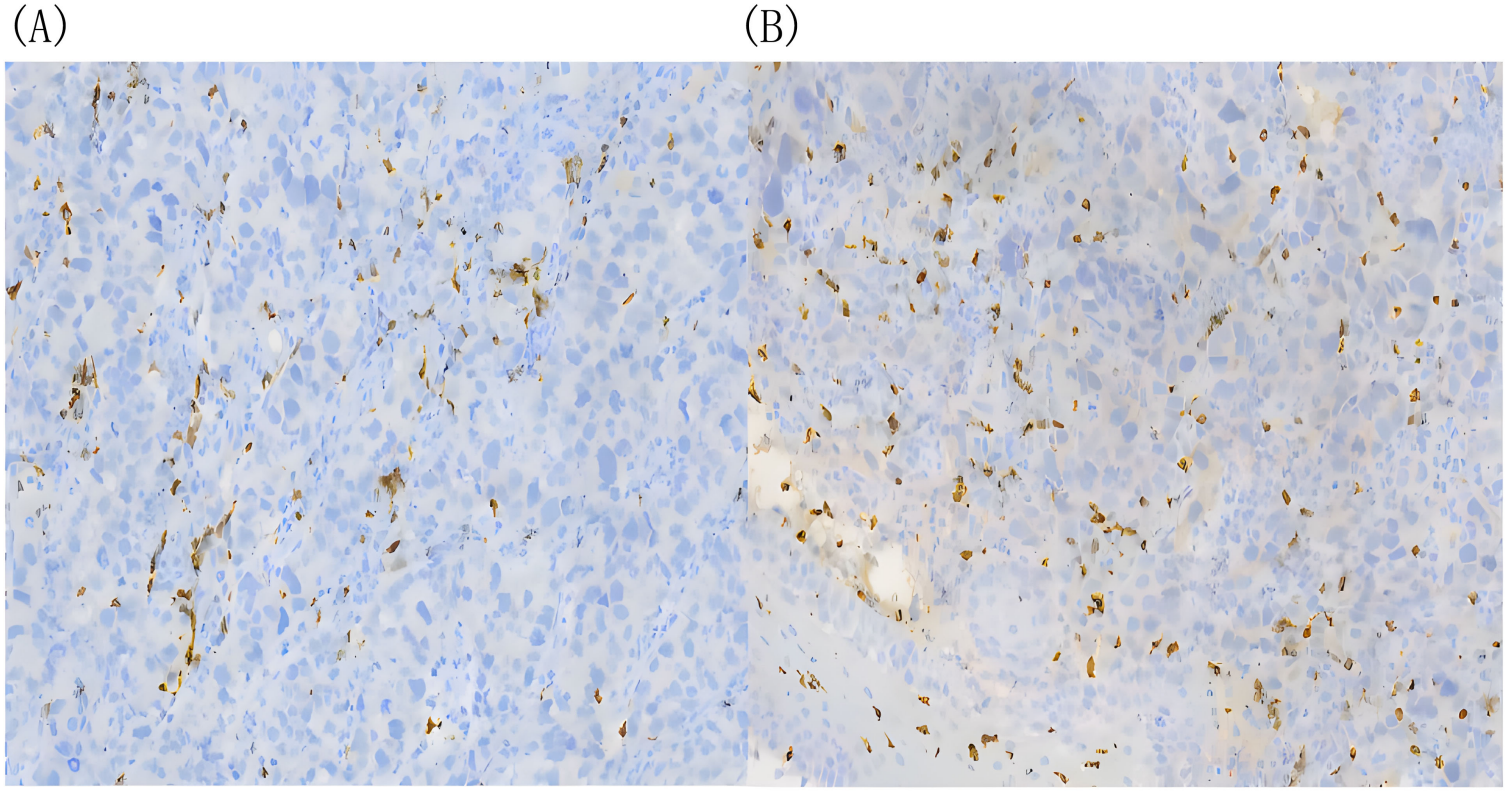
There was no significant difference in infiltrating CD8+ T cells before and after H101 treatment. **(A)** Before H101 treatment, the density of infiltrating CD8^+^ T cells in cardia lesion is low(+);**(B)** After H101 treatment, the density of infiltrating CD8^+^ T cells increased slightly (+). Reagent information: Rabbit monoclonal to CD8, Product code: ab217344, Abcam (Shanghai) Trading Co. LTD.

In February 2023, he was admitted to our department with an worsen eating obstruction. Chest and abdominal computed tomography (CT) scan showed cardia recurrence and multiple metastases in liver, peritoneum, abdominal cavity and retroperitoneal lymph nodes. Sintilimab (200mg, intravenously every three weeks) combined with nanoparticle albumin-bound paclitaxel(300mg, intravenously every three weeks) was admitted as first-line treatment after recurrence. After 4 cycles of chemotherapy plus ICI therapy, the patient´s metastases were significant shrink (**Figure 1**), but the cardia lesions were almost unchanged. As the patient strongly requested further relief of eating obstruction, we implemented exploratory local injection of H101 into recurrent cardia lesion by painless gastroscopy with the patient’s full knowledge and consent. Since June 9, 2023, H101 (5.0×10^11^ virus particles/0.5ml every time) was multipoint injected by painless gastroscopy every six weeks (8), 1 day before Sintilimab in each cycle. We observed on CT and gastroscopy images that the cardiac lesion were significantly reduced after two H101 treatment (**Figure 2**). Meanwhile, the patient´s eating obstruction symptom was subsequently relieved. Before and after two H101 treatment, we biopsied the cardia lesions and performed immunohistochemical staining of CD4+T cells and CD8+T cells respectively, results revealed a significant promotion of CD4^+^ T cell infiltration after H101 treatment (**Figure 3**). Unfortunately, no significant infiltration of CD8+T cells was found after H101 treatment (**Figure 4**). Then Sintilimab combined with S-1(60mg orally twice daily for 14 days) was admitted as maintenance antitumor therapy to date. (The timeline of treatments is shown in **Table 1**).

**Table 1:** This table provides detailed information about the patient’s medical history, including the timeline of treatments.

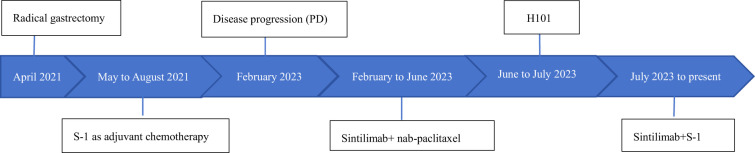

## Discussion

Here, We present a case involving an individual diagnosed with advanced cardia cancer who exhibited favorable responses to a combination therapy involving oncolytic virus, chemotherapy, and immunotherapy. We innovatively treated cardia cancer by local injection of oncolytic virus through gastroscopy and effectively reversed immune resistance. Following H101 treatment, the patient experienced notable reduction in cardia lesions, improvement in eating obstruction, and modest activation of the local tumor immune microenvironment, fostering infiltration of CD4+ T cells. Research in cancer immunotherapy has mainly focused on CD8+ T cells in the tumor microenvironment (TME), However, it has been reported that antitumor immunity cannot be induced unless the tumor cells have the MHC class II binding neoantigens, which are recognized by CD4+ T cells. CD4+ T cells are likely to be the driving force of the cancer immunity cycle, allowing for a continuous supply of cytotoxic lymphocytes(CTLs) to the TME. Additionally, studies also have demonstrated that after oncolytic viruses infect tumor cells, they induce cell lysis and the release of tumor antigens and other immunostimulatory molecules. While this process can trigger an immune response, these antigens and stimulatory factors may predominantly recruit and activate helper T cells (CD4+ T cells) rather than cytotoxic T cells (CD8+ T cells). CD4+ T cells play a crucial role in coordinating subsequent immune responses by secreting cytokines (9–11). This conclusion is consistent with our test results. No significant infiltration of CD8+T cells was observed after H101 treatment, possibly due to the deviation of the biopsy specimen location from the injection site, and lack of enough tissue samples to fully observe. The deeper reason may be due to the special characteristics of oncolytic virus to change the TME. Moreover, subsequent to the localized administration of H101, we observed effective control over distant liver metastases, thereby further substantiating the synergistic potential of oncolytic viruses in conjunction with immunotherapy.

Oncolytic viruses (OVs) represent a widely employed strategy for localized tumor treatment. Engineered to selectively target and destroy tumor cells while sparing normal tissue, they hold promise in cancer therapy. However, their clinical application remains constrained. After the approval of the herpes virus-based drug T-VEC by the US Food and Drug Administration (FDA) in 2015, other OVs such as adenovirus, coxsackie virus, and measles virus have since entered clinical investigation (12, 13). In a recent phase II clinical study, H101 also found to trigger a proliferative burst of CXCR6^+^ and GZMK^+^CD8^+^ T cells in malignant ascites (14). Nonetheless, OVs alone often fail to elicit robust therapeutic responses against malignant tumors, encountering obstacles like limited tumor penetration, constraints associated with local drug delivery, and pre-existing immune responses against the virus (15). These challenges underscore the need for innovative approaches to enhance the efficacy of OVs in cancer treatment.

To further enhance therapeutic efficacy, numerous genetically engineered oncolytic viruses have undergone development and clinical trials. A phase 1/2 clinical investigation demonstrated the feasibility and safety of LOAd703, an oncolytic adenovirus carrying transgenes encoding TMZ-CD40L and 4-1BBL, in combination with nab-paclitaxel and gemcitabine for treating patients with advanced pancreatic ductal adenocarcinoma (16). Additionally, efforts to diminish the extracellular matrix and facilitate oncolytic virus diffusion within tumors have led to modifications incorporating hyaluronidase or relaxin into oncolytic viruses, showing promising efficacy in preclinical studies (14, 17). In addition, to bolster dendritic cell maturation, GM-CSF fragments are integrated into viral genes to further stimulate immune activation (18).

Presently, immune checkpoint inhibitors demonstrate substantial effectiveness against advanced solid tumors (5, 19). Nonetheless, a majority of patients do not derive benefit, likely attributed to the heterogeneous nature of the tumor immune microenvironment. An expanding body of research indicates that modifying the tumor immune microenvironment holds promise for augmenting the effectiveness of immune checkpoint inhibitors and overcoming immune resistance (20, 21). Oncolytic viruses offer a compelling approach by efficiently lysing tumor cells to release antigens, inducing immunogenic cell death, fostering immune cell infiltration, and bolstering anti-tumor immunity (22). A preclinical study showed that oncolytic parapoxvirus ovis can induce GasderminE-mediated pyroptosis and activate anti-tumor immunity, adding new evidence for oncolytic viruses to stimulate anti-tumor immunity (23). A phase II trial found that H101 can trigger a proliferation burst of CXCR6^+^ and GZMK^+^CD8^+^T cells in malignant ascites, enhancing tumor-specific T cell cytotoxicity (14). Furthermore, biopsy results from another phase II trial, following treatment with a triple-mutated, third-generation oncolytic herpes simplex virus type 1, revealed an increase in tumor-infiltrating CD4^+^/CD8^+^ lymphocytes, while the count of Foxp3+ cells remained low (24). These findings suggest the effective activation of anti-tumor immunity by oncolytic viruses.

However, investigations into combining oncolytic viruses with immunotherapy for solid tumors predominantly reside in Phase 1/2 clinical trials, necessitating additional trials to conclusively establish the efficacy of this combination. This study underscores that oncolytic viruses, functioning as localized treatments, can manage primary lesion reduction and immune activation. Immunotherapy, serving as a systemic approach, can potentiate the cytotoxicity of immune cells and efficiently control lesions.

## Conclusion

We present a case study of a patient diagnosed with cardia cancer who underwent local administration of oncolytic virus H101 following ineffective treatment with paclitaxel combined with PD-1 inhibitors. Remarkably, this approach exhibited promising efficacy and immune-stimulating effects, offering insights into the selection of subsequent-line immunotherapy for cardia cancer. This finding unveils further therapeutic avenues for exploration.

## Data Availability

The original contributions presented in the study are included in the article/Supplementary Material. Further inquiries can be directed to the corresponding author.
